# Once-Weekly Liposomal Amphotericin B Use for Maintenance and Consolidation Phase Treatment of Cryptococcal Meningitis in Patients With AIDS

**DOI:** 10.7759/cureus.55824

**Published:** 2024-03-08

**Authors:** Vadim Belinschi, Charity Iheagwara, Ala Muhanna

**Affiliations:** 1 Internal Medicine, St. Michael's Medical Center, Newark, USA; 2 Infectious Disease, St. Michael's Medical Center, Newark, USA; 3 Infectious Diseases, St. Michael's Medical Center, Newark, USA

**Keywords:** immunocompromised, amphotericin b, cryptococcal meningitis, aids, hiv

## Abstract

Cryptococcal meningitis should be considered in individuals diagnosed with human immunodeficiency virus (HIV) infection and presenting with a cluster of differentiation 4 (CD4)-helper T cell count below 100 cells/ml. The 2022 guidelines from the World Health Organization (WHO) advocate for initiating treatment with a high dose (10 mg/kg) of liposomal amphotericin B, followed by flucytosine and fluconazole for a two-week duration. Additionally, alternative treatment options involving a combination of flucytosine and fluconazole are recommended. Consolidation therapy, as per the WHO guidelines, involves an eight-week course of fluconazole (800 mg), initiated after the induction phase. The dosage is then reduced to 200 mg/day, maintaining this level until the CD4 count exceeds 200 cells/mm^3^. Notably, the 2022 WHO guidelines prioritize a single dose of liposomal amphotericin B (LampB) over amphotericin B deoxycholate (AmpB-D) at 1 mg/kg due to its association with fewer side effects, including decreased mortality, kidney damage, and anemia. These recommendations are founded on the outcomes of the Ambisome Therapy Induction Optimization (AMBITION-CM), a multicenter, open-label, randomized controlled trial. This case report details the outpatient management of cryptococcal meningitis in a 47-year-old male with acquired immunodeficiency syndrome (AIDS) who exhibited intolerance to fluconazole. In this scenario, the decision to employ liposomal amphotericin B (LampB) as the sole agent for treatment during the outpatient phase was driven by challenges in tolerating fluconazole. Despite the absence of specific research on LampB's standalone use during the maintenance and consolidation phases, concerns regarding the patient's adverse reaction to fluconazole influenced the choice. Notably, LampB's once-weekly infusion schedule, although more expensive than AmpB-D, contributes to enhanced patient compliance. Exploring alternatives to traditional medications, such as interferon-gamma (INF-γ), Mycograb, 18B7, APX001, and T2307, holds promise in targeting novel antigens or complementing existing treatment regimens. Post-discharge, the patient received weekly LampB infusions alongside antiretroviral therapy (ART), resulting in an undetectable viral load and an increased CD4 count. A subsequent cerebrospinal fluid analysis post-discharge revealed a positive India ink stain but negative cultures for Cryptococcus, underscoring the necessity for a comprehensive and adaptable approach in managing cryptococcal meningitis.

## Introduction

Cryptococcal meningitis should be suspected in patients with human immunodeficiency virus (HIV) infection and a low cluster of differentiation 4 (CD4)-helper T cell count, usually less than 100 cells/ml. The World Health Organization’s 2022 guidelines recommend induction therapy with a high dose (10 mg/kg) of liposomal amphotericin B (LampB) followed by flucytosine and fluconazole for two weeks (other alternatives with combination of flucytosine and fluconazole are also recommended). For consolidation therapy, fluconazole (800 mg) is recommended for eight weeks during the induction phase and, for the consolidation phase, with a reduced dose (200 mg/day) until the CD4-helper T cell count reaches over 200 cells/mm^3^. It is important to emphasize that as per 2022 WHO guidelines, a single dose of liposomal amphotericin B (LampB) is the preferred agent (as opposed to amphotericin B deoxycholate (AmpB-D) 1 mg/kg), and it was associated with less side effects including a decrease in mortality, kidney damage, and development of anemia. These new recommendations were based on the Ambisome Therapy Induction Optimization (AMBITION-CM), a multi-center, open-label, randomized control trial. This case report describes a case of a 47-year-old male with acquired immunodeficiency syndrome (AIDS) who could not tolerate fluconazole which leads to the use of alternative LampB as a sole agent for outpatient management of cryptococcal meningitis.

## Case presentation

A 47-year-old male with a medical history of heterosexually acquired human immunodeficiency virus (HIV) presented to the emergency department with complaints of severe headache for eight days. He described his headache as intermittent and localized to the right occipital and neck region, exacerbated by cough. Associated symptoms included night sweats, anorexia, forgetfulness, nausea, and left shoulder pain. The rest of the review of systems was negative except for episodes of loose stools on the day of presentation. The patient's social and surgical history were unremarkable, and he never took any medications other than his antiretroviral therapy (ART). The patient had poor compliance with the ART therapy. The patient's vital signs on admission were blood pressure of 122/70 mmHg, heart rate of 67 beats per minute, respiratory rate of 14 breaths per minute, and oxygen saturation of 97%-100% on room air. Physical exam revealed a well-appearing male; positive findings included photophobia and neck stiffness on chin tuck. Bedside ophthalmoscopic evaluation did not reveal noticeable papilledema; there was no rash; and other than photophobia, there was no significant impairment in vision. Computer tomography of the head did not show any acute pathological process. Laboratory investigations within 24-48 h of admission were notable for an absolute CD4-helper T cell count of seven with leukopenia; the metabolic panel was unremarkable. Serum fungitell was normal. Kidney function was normal with blood urea nitrogen of 15 and serum creatinine of 0.9 (Table 1).

**Table 1:** Initial laboratory results within 24-48 hours of admission. AST: aspartate aminotransferase, ALT: alanine aminotransferase.

Component	Range	Results
Sodium	136-145 mmol/l	136 mmol/l
Potassium	3.5-5.3 mmol/l	4.3 mmol/l
Chloride	98-110 mmol/l	106 mmol/l
Bicarbonate	20-31 mmol/l	24 mmol/l
Anion gap	6-19 mmol/l	6 mmol/l
Blood urea nitrogen	6-24 mmol/l	15 mmol/l
Creatinine, serum	0.6-1.2 mmol/l	0.9 mmol/l
Glucose, serum	70-140 mmol/l	99 mmol/l
AST	10-36 mmol/l	19 mmol/l
ALT	9-46 mmol/l	21 mmol/l
Alkaline phosphatase	40-115 mmol/l	58 mmol/l
Total protein	6.4-8.4 g/dl	7.2 g/dl
Albumin	4.0-5.0 g/dl	4.2 g/dl
Phosphorus	2.1-4.3 mmol/l	2.7 mmol/l
Magnesium	1.5-2.5 mmol/l	1.9 mmol/l
White blood cells	4.4-11 x10^3^	3.6 x10^3^
Red blood cells	4.32-5.72 x10^6^	4.15 x10^6^
Hemoglobin	13-17.7 g/dl	13.8 g/dl
Hematocrit	37.5%-51%	39.4%
Platelets	150-450 x10^3^	151 x10^3^
Absolute CD4-helper T cell count	359-1519/μl	7/μl
Absolute CD8-suppressor T cell	109-897/μl	170/μl

**Figure 1:**
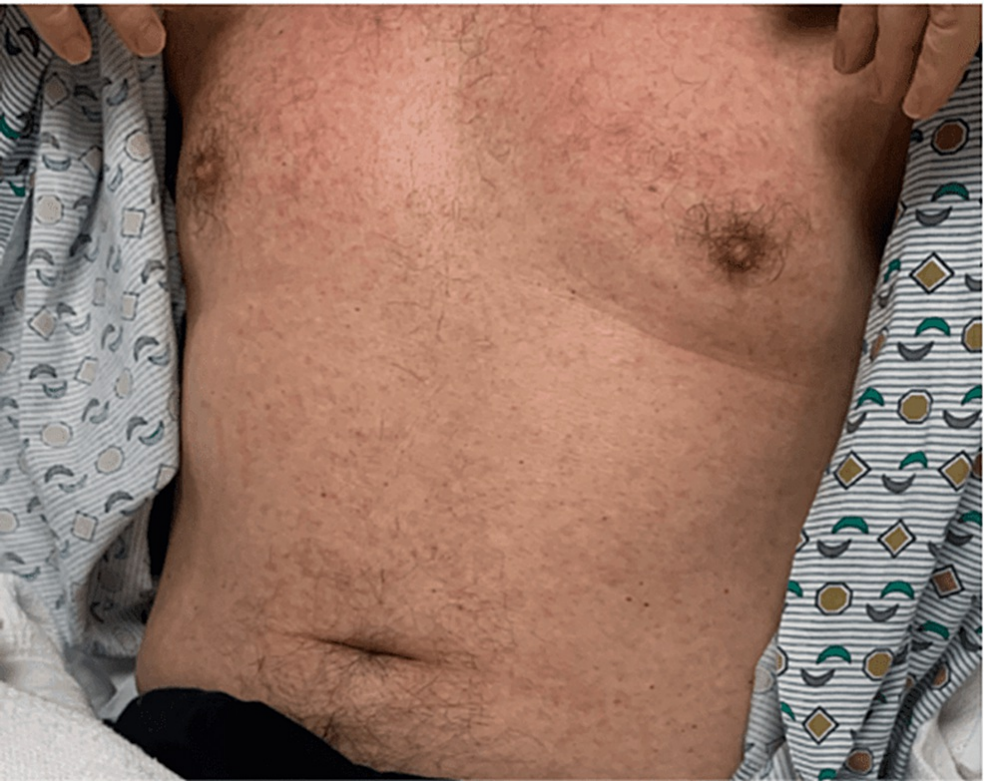
Patient developed a rash that was pruritic but resolved with steroids and discontinuation of fluconazole.

Computer tomography of the head was unremarkable. Subsequent blood cultures were positive for *Cryptococcus neoformans*. Cerebrospinal fluid (CSF) antigen with a positive titer of 1:128 dilution indicates a potentially significant infection. India ink stains of CSF fluid were positive as well. CSF culture was positive for yeast, *Cryptococcus neoformans*. CFS protein was elevated at 52.4, glucose was normal, and there were elevated white blood cells (WBC) with lymphocyte predominance, but no red blood cells (RBC) present. The patient had undergone a lumbar puncture on the day of the admission, three days after admission, and after two weeks inpatient. Initial opening pressure from the first lumbar puncture was not recorded; opening pressure was 22 cm of H_2_O on the second lumbar puncture and 32 cm of H_2_0 after two weeks. The patient was started on amphotericin-B (LampB) and fluconazole as per the recommended World Health Organization (WHO) 2022 guidelines. We opted for LampB due to its favorable side effects profile, as highlighted in the WHO 2022 guidelines [1,2]. Liposomal formulation of amphotericin is preferred as part of the induction regimen based on the Ambisome Therapy Induction Optimization (AMBITION-CM) trial [3,4] which showed similar efficacy of liposomal formulation with less chance of developing kidney damage. After the initiation of the treatment, the patient developed acute kidney injury (AKI) and persistent electrolyte derangements (hypomagnesemia and hypokalemia). In the view of AKI, LampB was switched to every other day. This patient's treatment was further complicated by the development of a full-body maculopapular blanching rash (Figure 1) along with fever, but there was no eosinophilia or eosinophiluria.

Rash and AKI were attributed to fluconazole which further complicated treatment options. Our team was concerned that the rash and AKI were due to Drug Reaction with Eosinophilia and Systemic Symptoms (DRESS syndrome). Eosinophilia was absent in this case. While rare, it is important to note that DRESS syndrome can manifest without eosinophilia, with reports suggesting its presence in only about one-third of cases [5-8]. The patient was started on intravenous fluid (IVF) therapy and steroids with careful monitoring and repletion of the electrolytes, and fluconazole was discontinued. The patient was maintained on every other day of LampB for 19 doses. With the help of IVF, his kidney function began to recover and magnesium and potassium remained stable. Repeat blood cultures were negative, and repeat cerebrospinal fluid analysis showed no growth after initiating therapy (Table 2). As per the patient’s clinical course, we chose not to follow the WHO 2022 guidelines regarding the maintenance and consolidation phase. Since the patient developed a reaction to fluconazole, a decision was made to discharge the patient on weekly doses of LampB for maintenance therapy. Outpatient therapy was maintained with once-weekly infusions of LampB. The patient's cerebrospinal fluid studies (Table 2) were compared during admission, after 72 hours of treatment, after 14 days, and after three months of outpatient treatment.

**Table 2:** Results of cerebrospinal fluid on admission, after 72 hours of initiating treatment, 14 weeks of inpatient treatment, and three months of outpatient treatment.

Component	Reference range	On admission	After 72h	After 14 days	Outpatient 3 months since discharge
Appearance spinal fluid	Clear	Clear	Clear	Clear	Clear
Opening pressure spinal fluid	18-20 cm H_2_O	Not recorded	22 cm H_2_O	32 cm H_2_O	18 cm H_2_O
Glucose spinal fluid	40-80 mg/dl	55 mg/dl	47 mg/dl	81 mg/dl	64 mg/dl
Protein spinal fluid	15-45 mg/dl	73 mg/dl	52 mg/dl	35 mg/dl	39.7 mg/dl
White blood cells spinal fluid	0.0-5.0/mm^3^	16/mm^3^	73/mm^3^	10/mm^3^	3.0/mm^3^
Lymphocytes % spinal fluid	%	96%	80%	89.0%	-
Monocytes % spinal fluid	%	1%	5%	5%	-
Neutrophils % spinal fluid	%	3%	15%	6%	-
Red blood cells	0.0-5.0/mm^3^	43/mm^3^	0.0/mm^3^	2.00/mm^3^	96.0/mm^3^
Antigen titer spinal fluid	Negative	1-128	1-256	-	1-256
India ink stain spinal fluid	Negative	-	Positive	Positive	Positive
Culture results spinal fluid	No growth	+ *Cryptococcus neoformans*	-	No growth	No growth

## Discussion

In our clinical case, we encountered several challenges that led us to opt for LampB as the primary agent for the maintenance and consolidation treatment. Notably, there is a paucity of research investigating the use of LampB as the solitary agent in this treatment phase. The WHO guidelines, in the absence of substantial data, continue to heavily lean on expert opinions. Our thorough exploration of PubMed did not uncover any studies that specifically investigated the utilization of LampB as a sole agent for the maintenance and consolidation treatment of cryptococcal meningitis.

Our decision to employ LampB as the sole outpatient maintenance agent, coupled with antiretroviral therapy (ART), stemmed from concerns about the patient's adverse reaction to fluconazole, which raised the possibility of a more severe response upon re-exposure. Despite LampB being somewhat more expensive than AmpB-D, it remains relatively cost-effective. Its once-weekly infusion schedule eases the burden on the patient and increases compliance. Although fluconazole is the preferred drug and has shown to be effective for maintenance therapy (as outlined in the WHO guidelines and expert opinion), we were limited to liposomal amphotericin B due to the aforementioned complications. Furthermore, alternative medications have been under investigation, showing promise in targeting novel antigens or working synergistically with current regimens. Some notable alternatives include INF-γ, currently undergoing a phase II trial; Mycograb, in phase II trials for its synergistic approach with amphotericin B; and 18B7, a monoclonal antibody targeting the capsular layer of Cryptococcal meningitis. Additionally, APX001, which inhibits the glycosylphosphatidylinositol biosynthesis pathway, is being evaluated either alone or in combination with fluconazole. T2307, a molecule acting on the mitochondria of *C. neoformans*, has exhibited promise in mouse studies. Sertraline, despite showing potential in vitro and murine models as an inhibitor of cryptococcal protein synthesis, did not demonstrate a reduction in mortality in phase III clinical trials. Lastly, tamoxifen, when used in combination with amphotericin B and fluconazole in murine models, exhibited a significant decrease in fungal burden in brain tissue, with ongoing research in this area [9]. Subsequent to discharge, meticulous surveillance of the patient's clinical trajectory was diligently conducted. The patient received LampB infusions on a weekly basis, constituting an extended outpatient therapeutic regimen spanning a total duration of 19 weeks; furthermore, the patient was initiated on ART (dolutegravir and lamivudine). Virological assessment performed one-month post-discharge demonstrated an undetectable viral load, equally noteworthy, an elevation in the patient's CD4 count, manifesting ascent from the initial nadir of 7 to 62 at seven months post-discharge. Repeat CSF three months post-discharge unveiled a positive India ink stain, albeit devoid of cellular presence, with protein and glucose concentrations well within normal parameters. CSF cultures were also negative for Cryptococcus. The utility of monitoring serum and cerebrospinal fluid cryptococcal antigen levels may be limited, particularly if they remain persistently positive over an extended period [10].

## Conclusions

In conclusion, the case presented here underscores the importance of individualized treatment approaches for patients with cryptococcal meningitis, particularly in the context of unique clinical challenges. While the 2022 WHO guidelines provide a valuable framework for the management of cryptococcal meningitis, they acknowledge the limitations in the evidence supporting maintenance and consolidation therapy, relying on expert opinion. Our decision to utilize liposomal amphotericin B (LampB) as the primary outpatient maintenance agent was driven by the patient's adverse reaction to fluconazole, highlighting the need for alternative treatment options. LampB, with its once-weekly infusion schedule, offers advantages in terms of patient compliance and quality of life, although it may be relatively more expensive than amphotericin B deoxycholate (AmpB-D). Furthermore, ongoing research into novel agents and combination therapies offers promising avenues for improving treatment outcomes in cryptococcal meningitis. These alternatives, such as INF-γ, Mycograb, 18B7, APX001, T2307, sertraline, and tamoxifen, represent potential future options for more effective and well-tolerated treatments. In light of this case and the evolving landscape of cryptococcal meningitis management, healthcare providers should remain vigilant, consider individual patient factors, and be open to exploring innovative approaches to optimize outcomes for this vulnerable patient population. Further research is needed to better define the role of LampB and other emerging therapies in the maintenance and consolidation phases, ultimately enhancing the care and prognosis of patients with cryptococcal meningitis.
